# An intrauterine device disappeared for nine years: a rare case report and literature review

**DOI:** 10.3389/fsurg.2026.1855066

**Published:** 2026-07-21

**Authors:** Jianfa Wu, Huang Chen, Qianyi Liao, Yuhua Zhang, Zhou Liu

**Affiliations:** 1 Department of Gynecology, Shanghai University of Medicine & Health Sciences Affiliated Zhoupu Hospital, Shanghai, China; 2 Department of Gynecology, Shanghai University of Medicine & Health Sciences, Shanghai, China; 3 Department of Integrated Traditional Chinese and Western Medicine Clinical Practice, Shanghai University of Traditional Chinese Medicine, Shanghai, China; 4 Department of Gynecology, Gongshan People’s Hospital, Nujiang Lisu Autonomous Prefecture, China

**Keywords:** bladder, case report, displacement, IUD, ultrasonic examination

## Abstract

**Background:**

The intrauterine device (IUD) displacement into the urinary bladder is an exceedingly rare complication. Given its potential to induce bladder irritation, erosion, or perforation, affected patients may present with lower abdominal pain, abdominal distension, and dysuria. As a result, clinical symptoms often prompt timely diagnosis, leading to early detection in the majority of reported cases.

**Case presentation:**

We report a rare case of IUD displacement into the urinary bladder that remained undiagnosed for nine years. Following her fourth IUD insertion, the patient developed lower abdominal pain, right lower limb edema, and impaired mobility; however, these symptoms were erroneously misdiagnosed during ultrasound examination. A definitive diagnosis was not established until nine years later, when she presented anew with persistent lower abdominal pain. Subsequent computed tomography (CT) scan and color Doppler ultrasound confirmed intravesical IUD migration, and the device was successfully retrieved via surgery.

**Conclusions:**

This case underscores the critical importance of thorough post-procedural evaluation, particularly in patients who develop new or persistent symptoms following IUD insertion. Furthermore, CT may be useful when ultrasound findings are inconclusive or when extrauterine displacement is suspected.

## 1 Background

The IUD is among the most effective and widely adopted long-acting reversible contraceptives globally, with an estimated 100 million users worldwide (1). In China alone, IUD use accounts for approximately 80 million individuals, representing nearly 40% of women of reproductive age (2).

However, the widespread adoption of IUDs is associated with a well-documented spectrum of complications, including abnormal uterine bleeding, pelvic inflammatory disease, uterine perforation, device embedment, partial or complete expulsion, and displacement into adjacent anatomical structures (3). Notably, although IUD displacement is rare, it carries substantial clinical risk, potentially leading to secondary injury to the uterus, bowel, bladder, ureters, kidneys, or even the stomach (4, 5).

Among these complications, IUD displacement into the bladder is an exceptionally rare event. Affected individuals may present with lower abdominal pain, abdominal distension, and dysuria (6, 7). However, a small number of cases remain undiagnosed for prolonged periods due to the paucity or absence of characteristic symptoms, posing diagnostic challenges and potentially delaying intervention.

This manuscript presents a case of bladder-localized IUD with a remarkable nine-year diagnostic delay, masked by an atypical presentation that confounded initial clinical assessment.

## 2 Case description

**Figure 1:**
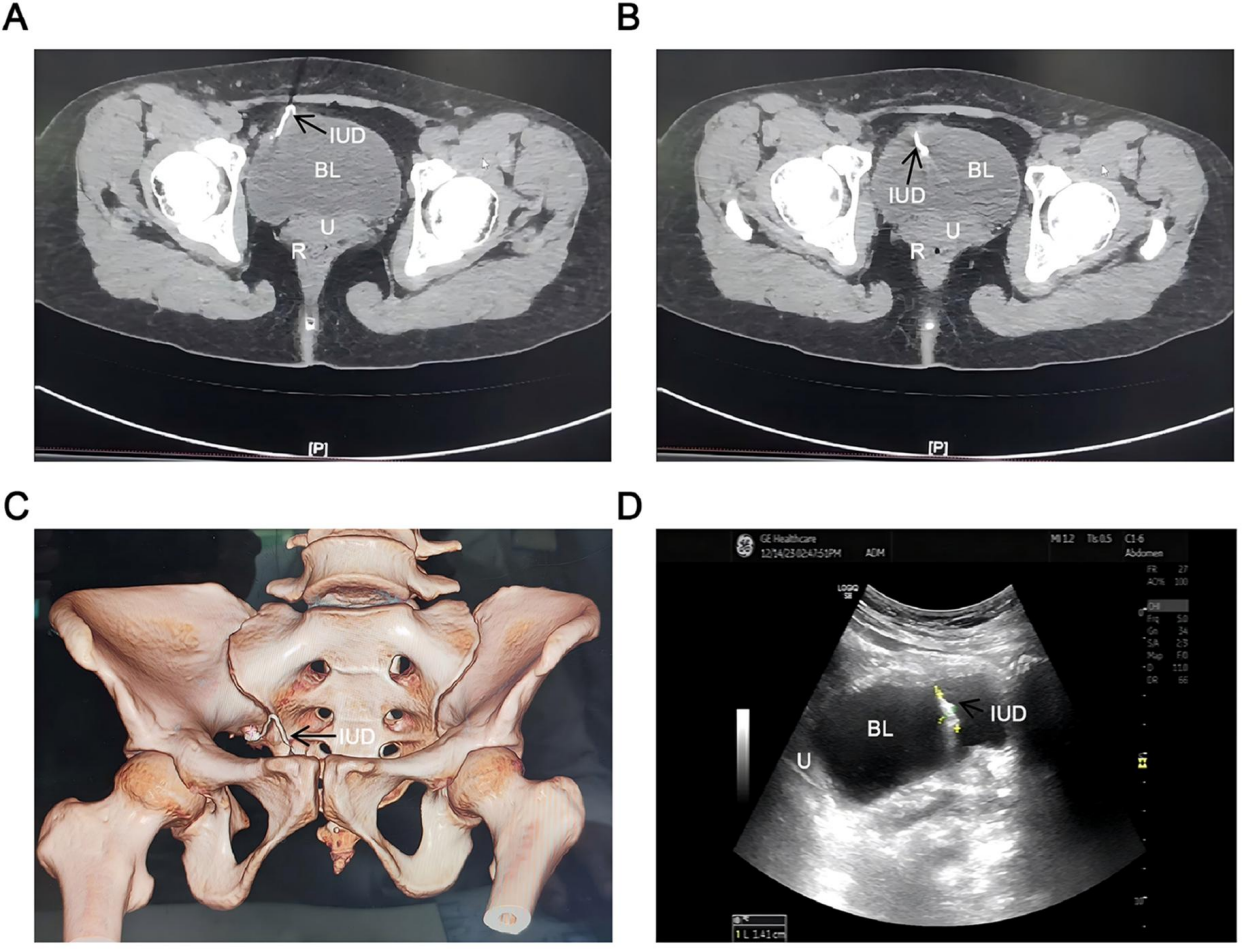
Assessment of the relationship between the IUD and the bladder before operation. **(A)** and **(B)** The relationship between the IUD and the bladder, uterus, and rectum was assessed using CT imaging; **(C)** The position relationship between the IUD and pelvis was evaluated by three-dimensional stereoscopic imaging; **(D)** The position relationship between the IUD and bladder was evaluated by color ultrasound. BL, bladder; U, uterus; R, rectum; IUD, intrauterine device.

A 38-year-old woman was admitted for evaluation of persistent lower abdominal pain lasting one week. CT examination and three-dimensional reconstructed imaging demonstrated metallic densities within the urinary bladder, with the largest fragment embedded in the bladder wall (Figures 1A–C). Color Doppler ultrasound identified a hyperechoic structure measuring 15*6 mm^2^ within the bladder lumen (Figure 1D). Notably, the patient denied urinary symptoms, including frequency, urgency, or painful urination, and reported no systemic signs, such as fever, chills, or hematuria. Physical examination revealed mild tenderness on palpation of the lower abdomen, without rebound tenderness or muscular guarding. Routine complete blood count and C-reactive protein (CRP) levels were within normal limits.

**Figure 2:**
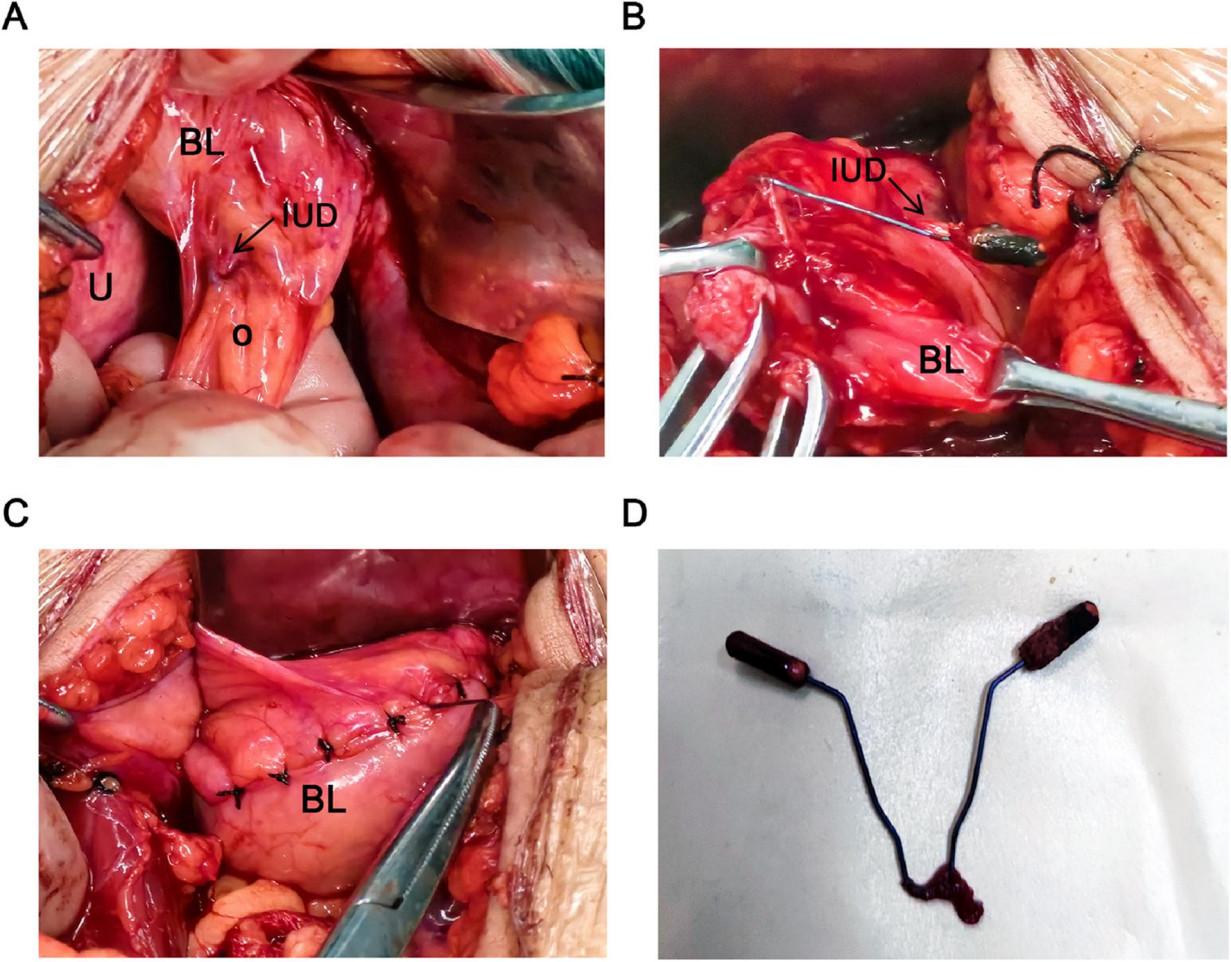
Assessment of the relationship between the IUD and bladder during operation. **(A)** The omentum encloses the IUD and is attached to the bladder; **(B)** A functional MYCu IUD was observed, with one arm embedded in the muscular layer and the other arm penetrating through into the lumen of the bladder; **(C)** An image of a sutured bladder; **(D)** An image of the removed MYCu IUD. B, bladder; O, omentum; U, uterus; IUD, intrauterine device.

Given the confirmed bladder displacement of the IUD and the absence of on-site laparoscopic surgical equipment, an open laparotomy was performed following written informed consent. Intraoperatively, dense adhesions between the greater omentum and the bladder were identified. Careful dissection revealed the IUD displaced across the bladder wall (Figure 2A). Detailed inspection confirmed a fully intact MYCu IUD: one arm was firmly embedded within the bladder muscle layer, while the contralateral arm traversed the full thickness of the bladder wall and displaced into the lumen (Figures 2B–D). The device was successfully extracted, followed by two-layer closure of the cystorrhaphy site with absorbable sutures.

The patient has undergone four IUD insertions, as summarized in Table 1. Her obstetric history includes six pregnancies: four term deliveries and two induced abortions, as outlined in Table 2.

**Table 1:** History of IUD insertion.

Sequence of IUD insertion	Time of IUD insertion	Type of IUD	Hospital of IUD insertion	Professional Title of the Operating Surgeon	Removal mode	Time of IUD removal	Follow-up examination after IUD removal	Symptom onset
1	2012–02	Gongxing	Gongshan Maternal and Child Health Hospital	Resident doctor	Spontaneous expulsion	2012–03	After the expulsion of an intrauterine device, re-examination via color Doppler ultrasound showed no intrauterine device residual in the uterine cavity, and no abnormalities were detected in the uterus and adnexa.	Asymptomatic
2	2012–07	Gongxing	Gongshan Maternal and Child Health Hospital	Attending doctor	Spontaneous expulsion	2012–09	The patient did not return to the hospital for re-examination after the intrauterine device was expelled due to personal reasons.	Asymptomatic
3	2013–03	Copper T	Gongshan Maternal and Child Health Hospital	Attending doctor	Spontaneous expulsion	2013–05	After the expulsion of an intrauterine device, re-examination via color Doppler ultrasound showed no intrauterine device residual in the uterine cavity, and no abnormalities were detected in the uterus and adnexa.	Asymptomatic
4	2014–03	MYcu	Gongshan Maternal and Child Health Hospital	Resident doctor	Uknown	Unknown	In April 2014, she presented with abdominal pain, swelling of the right lower limb, and reduced mobility. Re-examination via color Doppler ultrasound showed no intrauterine device residual in the uterine cavity, and no abnormalities were detected in the uterus and adnexa. After a day of rest, her symptoms improved.	Abdominal pain, swelling of the right lower limb, and reduced mobility

**Table 2:** History of pregnancy.

Time	Pregnancy outcome	Method of delivery or miscarriage	Postpartum complications
2008–06 2010–10 2017–03 2020–01 2021–07 2023–02	Term delivery Term delivery Term delivery Second-trimester induced abortion Early induced abortion Term delivery	Vaginal delivery Vaginal delivery Vaginal delivery Vaginal delivery Vaginal delivery Vaginal delivery	No No No No No No

**Figure 3:**
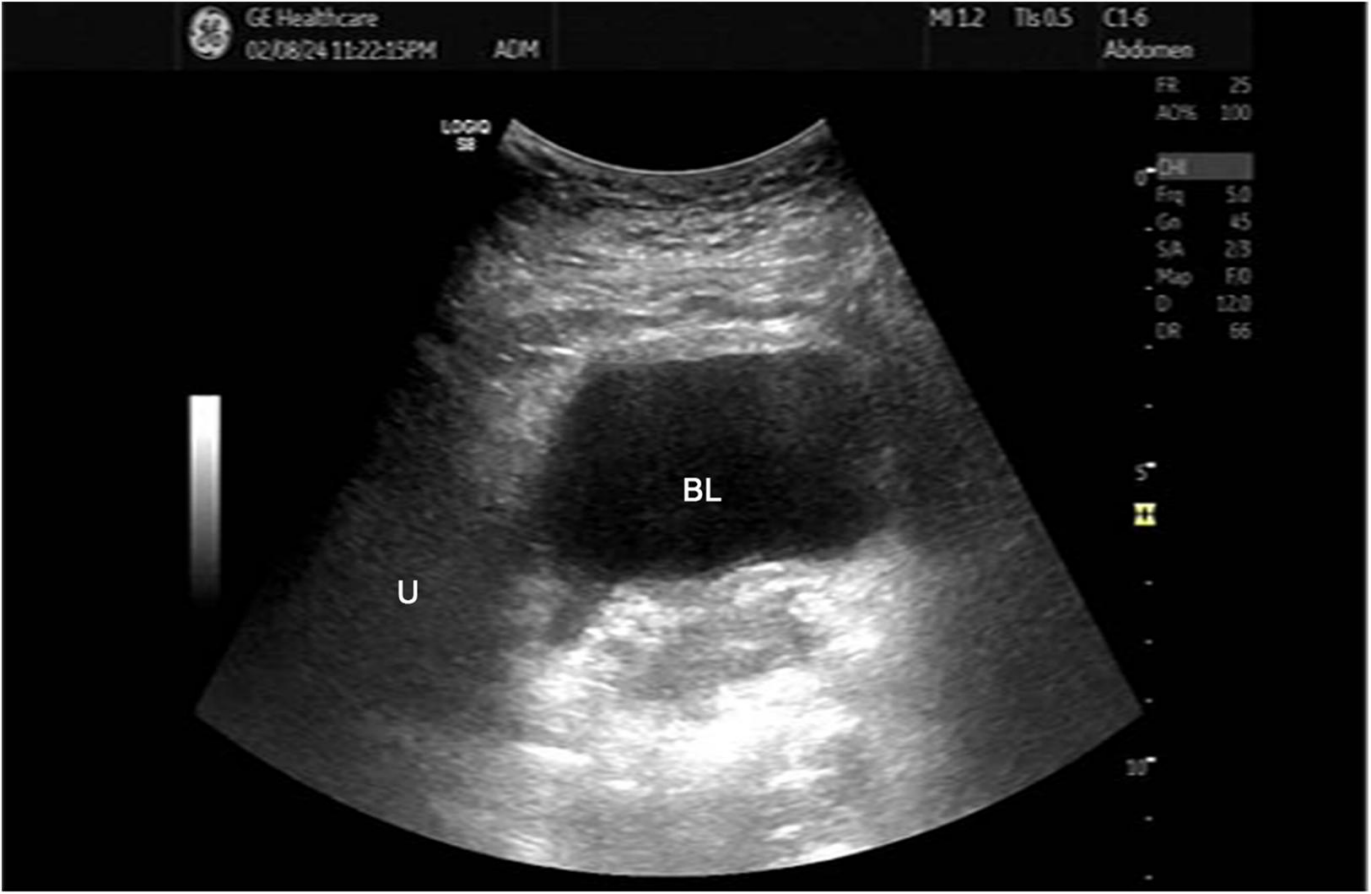
Postoperative bladder re-examination. The bladder was evaluated by color ultrasound. U, uterus; BL, bladder.

The patient reported no prior surgical procedures or chronic medication use. Postoperatively, she experienced an uneventful recovery and was discharged on postoperative day 5. A follow-up ultrasound performed on February 8, 2024, demonstrated normal bladder wall architecture and luminal contour, with no evidence of intravesical foreign material or structural abnormality (Figure 3). The patient remained asymptomatic with respect to lower urinary tract function. Specifically, no urinary frequency, urgency, dysuria, or hematuria was reported.

## 3 Literature review methodology

A systematic literature search was conducted in PubMed to identify studies published until December 31, 2025, that reported cases of IUD displacement into the bladder or intravesical space. The search strategy employed the following Boolean combination: (“Intrauterine Device” OR “IUD”) AND (“bladder” OR “intravesical” OR “migration”). Following removal of duplicates, 522 records were retrieved. Full-text assessment was then performed to confirm inclusion criteria, and backward citation tracking of eligible articles was conducted to identify additional relevant studies. After applying predefined exclusion criteria, including case reports without imaging confirmation, and studies lacking clear documentation of IUD displacement into the urinary bladder, a total of 30 studies met all inclusion criteria and were included in the final analysis.

## 4 Discussion

Uterine perforation is a rare but potentially serious complication of IUD insertion, with reported incidence rates ranging from 0.3% to 2.6% (6–9). Established risk factors include the postpartum and lactational states, operator inexperience, and anatomical uterine variants such as retroflexion or congenital malformations (10–13). Notably, lactation at the time of insertion confers a six-fold increased risk of perforation compared with non-lactating states (10). Following perforation, intraperitoneal adhesions, most commonly involving the greater omentum and small bowel, are the predominant sequelae. IUD displacement is a well-recognized consequence of uterine perforation; documented ectopic locations include the stomach, rectum, kidney, and urinary bladder (14–18). The patient had no prior history of uterine perforation. All four IUD insertions were performed outside the postpartum lactation period. Each procedure was conducted at a primary-level maternal and child health care facility: two by resident physicians and two by attending physicians. Given the absence of prior uterine trauma and the timing of insertion relative to lactation status, the current case of IUD displacement into the bladder is most plausibly attributable to procedural error during insertion.

Transvaginal and transabdominal ultrasound are routinely employed for IUD surveillance. However, their sensitivity in diagnosing IUD displacement is limited. Notably, color Doppler ultrasound, commonly used for post-insertion follow-up, has demonstrated a high false-negative rate for displaced IUDs, with reported missed diagnosis rates reaching 28% (Table 3) (11, 19–23). In contrast, CT offers superior spatial resolution and tissue characterization, making it highly effective for confirming displaced IUD localization (25–27). CT imaging can reliably identify cases of IUD displacement overlooked by ultrasound, thereby mitigating diagnostic delay and preventing inappropriate clinical management (11, 19, 21, 22). Additionally, abdominal radiography (plain film) also serves as a valuable adjunctive tool, particularly in resource-limited settings or when rapid initial screening is indicated, as it readily detects radiopaque IUDs regardless of anatomical location (27–29). The present case illustrates a 9-year diagnostic delay in identifying intravesical IUD displacement, attributable to an initial ultrasound misinterpretation. This underscores the critical importance of maintaining a high index of suspicion for IUD displacement in patients presenting with persistent or atypical symptoms following insertion, and selecting confirmatory imaging modalities accordingly.

**Table 3:** Studies on IUD displaced in bladder.

Authors	Year	Number of cases	Age	Symptom	Duration of IUD placement(Months)	IUD removal method	Type of IUD	CT scan	Doppler ultrasound	Plain film
Heaney C et al. (10)	2023	1	40	Recurrent urinary tract infections, dysuria, right flank pain radiating to the groin, associated fever, nausea, myalgias, urinary urgency, frequency, hematuria, and malodorous urine	Unknown	Cystoscopy and robot-assisted Laparotomy	Copper T	Success	-	-
Liu G et al. (11)	2021	2	37	Urinary frequency, urgency, and hematuria	108	Laparotomy	MYCu	Success	Failed	-
			46	Frequent urination, urgency and pain	132	Laparotomy	Primary ring	Success	Failed	-
Xiong BJ et al. (32)	2020	11	34.45±1.92	Abdominal pain (4)	38.55±9.62	Laparoscopy (6)	MYCu(5)	-	-	-
				Hematuria (4)		Laparotomy (5)	Copper T(1)			
				None (4)			Gynefix(2)			
							Multiload Cu(1)			
							Unknown(2)			
Cheung ML et al. (19)	2018	1	34	Vaginal drainage	Unknown	Laparoscopy	Copper T	Success	Failed	-
Bakri S et al. (24)	2023	1	54	Frequent urination, pain in urination and intermittency with position changing	84	Cystoscopy	Copper T	Success	-	-
Wan L et al. (30)	2021	4	39.2 ±6.10	Urinary pain, long and frequent micturition, dysuria, hematuria visible, and urinary urgency Urinary frequency, urgency, and pain lower abdominal pain and dysuria	186±59.70	Cystoscopy(2) Laparoscopy(1) Laparoscopy+Cystoscopy(1)	MYCu(4)	Success (2)	Success (2)	-
Yu HT et al. (25)	2022	1	44		84	Laparoscopy	MYCu	Success	-	-
Rasekhjahromi A (36)	2020	1	43	Suprapubic pain	60	Laparotomy	Copper T	-	Success	-
Niu H et al. (39)	2019	1	57	Hypogastric pain, pollakiuria, and burning of micturition	312	Nephroscope transurethrally	Copper T	-	Success	-
Chakir Y et al. (31)	2021	1	50		Unknown	YAG Holmium Laser and transurethral resectoscope	Copper T	Success	Success	-
Zakin D (33)	1984	8	26.14±6.01	Dysuria, burning on urination, frequency, nocturia, occasional hematuria, and lower abdominal and suprapubic pain	44.14±29.69	Laparotomy (4) Cystoscopy (3) Vaginal Cystotomy (1)	Lippes loop (5) Dalkon shield (1) Copper T(2) MYCu	-	Success(2/2)	-
Chai W et al. (38)	2017	1	26	Urinary frequency and urgency, hematuria, and waist and abdomen pain	72	Cystoscopy		-	Success	-
Voulgaris AM et al. (40)	2015	1	30	Dysuria, intermittent visible haematuria and recurrent urinary tract infections	12	Cystoscopy	Unknown	-	Success	-
Kallat A et al. (41)	2017	1	40	Pelvic pain and straight back pain associated with intermittent haematuria and urination burns	144	Laparotomy	Copper T	Success	Success	-
Hosseinkhani A et al. (20)	2018	1	35	Recurrent vaginal infection, urinary tract infection, hesitancy, and suprapubic discomfort	168	Laparoscopy	Copper T	-	Failed	-
Rasyid N et al. (26)	2021	1	30	Suprapubic pain and dysuria	1	Cystoscopy	Copper T	Success	-	-
Vural M et al. (42)	2010	1	48	Pelvic pain	180	Cystoscopy+Laparoscopy	Copper T		Success	-
Christodoulides AP et al. (37)	2020	1	67	Dysuria, pelvic pressure, suprapubic and pelvic pain	240	Cystoscopy	MYCu	-	Success	-
Yahsi S et al. (21)	2015	1	37	Suprapubic pain, polyuria, and urgency	72	Cystoscopy+Laparoscopy	Copper T		Failed	-
Han X et al. (18)	2021	1	40	None	36	Hysteroscopy+cystoscopy	MYCu	Success	Success	-
Salih MA et al. (22)	2022	1	38	Gross hematuria, lower abdominal pain, and storage lower urinary tract symptoms	180	Laparotomy	Copper T	Success	Failed	-
Zhang NN et al. (43)	2020	1	39	Frequent urination	2	Hysteroscopy+cystoscopy		Success	-	-
Ozçelik B et al. (45)	2003	1	28	recurrent urinary infection and lower abdominal pain	6	Cystoscopy	Copper T	Success	-	Success
Ni D et al. (27)	2023	1	41	Frequent, urgent and painful urination	240	Cystoscopy	Copper T		Success	Success
Zhang M et al. (34)	2023	13	32.77±5.44	Abdominal pain(5) Hematuria(1) Dysuria(1) Frequent urination(4) Painful urination(3) Urgent urination(2) Menopause(3) Asymptomatic(2)	50.54±43.47	Cystoscopy(4) Cystoscopy+Laparoscopy(2) Laparoscopy(2) Laparotomy(4) Cystoscopy+Laparotomy(1)	Copper T(5) HCu280 (2) MCu(1) MYcu(1) Medicated-*γ*(1) GyneFix(1) Medicated copper ring 165 (2)	-	-	-
Saputra AND (28)	2023	1	30	Urinary tract infection and dysuria	60	Laparoscopy+hysteroscopy	Copper T	-	Success	Success
Basiri A et al. (29)	2019	1	39	Recurrent urinary tract infection, dysuria and frequency	132	Cystoscopy	Copper T	-	Success	Success
Makary J et al. (23)	2021	1	Unknown	Urinary frequency and dysuria	96	Cystoscopy	Copper T	-	Failed	-
Vahdat M et al. (44)	2019	1	31	Urinary tract infection	96	Cystoscopy+hysteroscopy	Copper T	-	Success	Success
Raj A et al. (35)	2021	1	28	Pelvis discomfort, and urinary tract infection	72	Cystoscopy+hysteroscopy	Unknown	Success	Success	Success

The principal clinical manifestations of IUD displacement include frequency, urgency, dysuria, hematuria, recurrent or persistent urinary tract infection (UTI), vaginal drainage, and lower abdominal or pelvic pain. Among these, UTI and lower abdominal/pelvic pain are the most commonly reported symptoms (Table 3) (10, 30, 31). In the present case, the predominant symptom was chronic lower abdominal discomfort, consistent with the established symptom profile in the literature. Consequently, IUD displacement should be actively considered in any patient presenting with new-onset, recurrent, or treatment-refractory lower urinary or pelvic symptoms following IUD insertion.

IUD displacement typically occurs within 1 to 312 months following insertion: cumulative incidence rates of 25% within the first year, 6.25% between years 2–4, 27.08% between years 5–7, 14.58% between years 8–10, and 27.08% beyond 10 years (Table 1) (32–35). These data indicate a biphasic temporal pattern of risk, characterized by an initial peak within the first year and a secondary peak after 5 years, rather than a monotonic increase or decline over time.

IUDs implicated in bladder displacement include several widely used devices, including MYcu, Copper T, HCu280, Medicated-gamma, GyneFix, and Medicated copper ring 165 (Table 3) (32, 34, 36–38). Among these, MYcu and Copper T are the most frequently reported. The present case, featuring an intravesical MYCu device, further corroborates existing evidence in the literature. This predominance likely reflects both higher global utilization rates of these two devices and their structural characteristics: specifically, the presence of rigid, non-resorbable side arms, which may increase mechanical stress on the uterine wall during insertion or subsequent uterine contractions, thereby elevating the risk of perforation and subsequent bladder displacement.

Surgical intervention is the standard of care for confirmed IUD displacement. Multiple minimally invasive and open approaches have been described, including laparoscopy, laparotomy, cystoscopy, laparoscopy combined with cystoscopy, transurethral nephroscope procedures, YAG Holmium Laser and transurethral resectoscope techniques, hysteroscopy combined with cystoscopy, laparoscopy combined with hysteroscopy, and cystoscopy combined with laparotomy (Table 3) (39–45). Currently, the majority of reported interventions have achieved successful device extraction with minimal morbidity. One exception involved transient urinary leakage following cystoscopy combined with holmium laser treatment, which was subsequently resolved by laparoscopic cystorrhaphy (32). The selection of surgical modality is guided by three key factors: (1) the precise anatomical location and depth of IUD embedding within the bladder wall; (2) evidence of concomitant involvement of adjacent structures (e.g., omentum, bowel); (3) institutional expertise and available resources. In this particular case, due to the absence of laparoscopic equipment, laparotomy was taken into account, resulting in complete IUD removal and primary two-layer bladder repair without complication.

In conclusion, intravesical IUD displacement is a rare but clinically significant complication. Although infrequent, its potential for long-term urological morbidity must not be underestimated. Clinicians should maintain a high index of suspicion in patients presenting with new-onset, persistent, or treatment-refractory lower urinary or pelvic symptoms following IUD insertion. Moreover, asymptomatic displacement, though uncommon, has been documented; thus, structured follow-up remains essential. Prior to surgical management, comprehensive preoperative assessment is mandatory, which includes precise localization of the IUD (e.g., via CT), evaluation of embedding depth and adjacent organ involvement (e.g., omental or bowel adhesions), and determination of institutional procedural capacity. The choice of surgical approach must be individualized to optimize complete device extraction while minimizing iatrogenic injury and preventing complications such as urinary leakage or bladder perforation.

## Data Availability

The original contributions presented in the study are included in the article/supplementary material, further inquiries can be directed to the corresponding author/s.
